# Feasibility of Multimodal Energy-Based Therapy for Pelvic Floor Disorders

**DOI:** 10.3390/medicina61122078

**Published:** 2025-11-21

**Authors:** Yoav Baruch, Clarissa Costa, Marta Barba, Alice Cola, Matteo Frigerio

**Affiliations:** 1 Urogynecology and Pelvic Floor Unit, Lis Hospital for Women’s Health, Sourasky Medical Center, Gray Faculty of Medical & Health Sciences, Tel Aviv University, Tel Aviv-Yafo 6997801, Israel; 2 Department of Obstetrics and Gynecology, ASST Monza, San Gerardo Hospital, University Milano-Bicocca, 20900 Monza, Italy; c.costa14@campus.unimib.it (C.C.); m.barba8792@gmail.com (M.B.); alice.cola1@gmail.com (A.C.); frigerio86@gmail.com (M.F.)

**Keywords:** pelvic floor dysfunction, urinary incontinence, dyspareunia, laser, photobiomodulation, radiofrequency, electrotherapy, electroporation

## Abstract

*Background and Objectives*: Pelvic floor disorders are highly prevalent among women globally and can severely compromise daily functioning and well-being. Emerging energy-based modalities have reshaped conservative management strategies, by allowing individualized therapeutic approaches. The aim of this study was to evaluate the utility of customized energy-based applications, via an innovative multimodal EVA/DAFNE device that incorporates multimodal energy-based synergistic technologies for the treatment of pelvic floor dysfunction. *Materials and Methods*: Patients with PFDs (pelvic organ prolapse, all types of urinary incontinence, bladder voiding dysfunction, and dyspareunia) who selected conservative treatments were prospectively enrolled. Baseline and after-treatment quality of life was assessed using the following validated tools: Urinary Distress Inventory-6 (UDI-6), Pelvic Organ Prolapse Distress Inventory-6 (POPDI-6), Female Sexual Function Index-6 (FSFI-6), Marinoff Scale, 0-100 VAS, and Vaginal Health Index. Overall improvement was measured through the Patient Global Impression of Improvement (PGI-I). Three to five sessions of treatment tailored according to the patient’s symptoms and clinical findings were delivered. Data were analyzed using standard statistical methods. *Results*: Twenty-six women with PFD who desired energy-based conservative treatment were recruited. Mean age was 48.6 ± 16.7 years. Indications for treatment were dyspareunia (*n* = 10; 38.5%), stress urinary incontinence (*n* = 9; 34.6%), mild pelvic organ prolapse (*n* = 6; 23.1%), genitourinary syndrome of menopause (*n* = 5; 19.2%), voiding dysfunction (*n* = 4; 15.4%), and overactive bladder syndrome (*n* = 2; 7.7%). Mean number of treatments was four. Baseline and after-treatment quality-of-life scores differed significantly. According to PGI-I scores 88.5% of patients considered themselves improved. *Conclusions*: Our study provides pilot estimates as to the safety and efficacy of a multimodal integrated treatment protocol for the treatment of PFD. Integrating multimodal energy-based conservative therapy into tailored treatment protocols can prove efficient and useful.

## 1. Introduction

Pelvic floor dysfunctions (PFDs) are highly prevalent conditions, affecting up to 50% of middle-aged and older women [1,2]. The main pelvic floor dysfunctions (PFDs) are pelvic organ prolapse (POP), urinary incontinence (UI), overactive bladder syndrome (OAB), fecal incontinence, and sexual dysfunction. Urinary incontinence is the most common type of PFD, affecting one in three women. Its two main types are stress urinary incontinence (SUI) and urge urinary incontinence (UUI). SUI is characterized by involuntary urine leakage triggered by a rise in intra-abdominal pressure. UUI manifests as involuntary leakage accompanied by a strong sensation of urgency and an immediate voiding that cannot be delayed. Approximately one in nine women is affected by POP, a condition defined as the descent of one or more pelvic organs from their anatomical position, resulting in bulging into the vagina [1,2,3]. These disorders significantly affect women’s quality of life, often leading to physical discomfort, psychological distress, social limitations, and low self-esteem [4].

The development of PFD in most cases is attributable to birth-related pelvic floor injury, while other contributing risk factors include age, and high BMI. The pathophysiology is multifactorial and involves genetic and hormonal factors, alterations in connective tissue support, as well as inherent inflammatory and proliferative mechanisms. The pelvic organs are supported by muscles and connective tissues that ensure their proper placement and functioning. Changes in the connective tissue of the urogenital tract, both qualitative and quantitative, occur primarily as a result of atrophy, degeneration, necrosis, and reduced collagen production associated with hormonal deprivation, ultimately leading to weakening of the urogenital support structures. In addition, impaired pelvic connective tissue strength has been linked to alterations in extracellular matrix metabolism, loss of key cell populations (such as fibroblasts, muscle and peripheral nerve cells), as well as oxidative stress and chronic inflammation [5]. The vaginal wall is composed of a superficial layer of non-keratinized squamous epithelium with high water content, supported by connective tissue, smooth muscle, collagen, and elastin that provide strength and elasticity. With age, declining estrogen levels reduce collagen and elastin production, leading to thinning of the vaginal wall, decreased elasticity, and dryness [6].

Treatment of PFD is mainly conservative and symptom-based, aiming to strengthen and support the pelvic floor. Management options include lifestyle and behavioral therapy, pelvic floor muscle training (PFMT), pharmacological treatment, medical devices, and surgery [7]. PFMT improves strength, endurance, power, and relaxation of pelvic muscles, and is recommended as the first-line therapy for PFD [8]. Biofeedback increases patients’ awareness and plays an important role in the treatment of PFD, and combining perineal muscle strengthening with biofeedback enhances somatosensory sensitization [9,10]. In women diagnosed with OAB after menopause the use of local estrogen therapy proved beneficial for improving urinary symptoms [11]. As for invasive interventions, these are not recommended unless other treatments prove ineffective.

Recently, energy-based technologies (electrotherapy, photobiomodulation, radiofrequency, and electroporation) have emerged as promising adjunctive or alternative conservative treatments for PFD. These modalities differentially enhance tissue regeneration, neuromuscular function, and vaginal health parameters. Electrotherapy engages controlled electrical stimulation, lower than 1000 μA, to enhance passive activation of muscle fibers and nerves [12,13]. Radiofrequency uses controlled thermal energy to induce collagen remodeling and neocollagenesis.

Lin et al. demonstrated that eight weeks of extracorporeal shock wave therapy could improve SUI symptoms during physical activity, reduce OAB symptoms, and promote quality of life [14]. Pulsed magnetic stimulation has been reported effective for the treatment of urinary incontinence [15,16]. High frequency electromagnetic waves that generate heat temperatures of 40–45 °C upon meeting tissue impedance induce collagen production, by activating fibroblasts and instigating an inflammatory cascade, and thereby seemingly improve UI, reduce pelvic pain, recover pelvic floor muscle strength, and advance sexual function [17]. Electroporation manipulates high-intensity electrical pulses to transiently increase cell membrane permeability, allowing enhanced transport of ions and molecules, thereby promoting cellular repair and improving tissue oxygenation [18].

Photobiomodulation (PBM), formerly known as low-level laser therapy (LLLT), refers to the use of non-ionizing monochromatic (or quasi-monochromatic) light in the visible and infrared spectrum for medical applications [19,20,21,22]. It employs low-dose lasers or light-emitting diodes (LED), absorbed by endogenous chromophores, to stimulate mitochondrial cytochrome C oxidase, thereby increasing ATP synthesis, modulating reactive oxygen species (ROS), and activating transcription factors that drive protein synthesis, collagen production, cell proliferation, and tissue regeneration [19,20,21,22]. Red (620–700 nm) and near-infrared (700–1440 nm) light stimulate fibroblasts, enhance collagen and elastin synthesis, and promote tissue repair, while blue light (400–500 nm) modulates proliferation, supports wound healing, and exerts antimicrobial effects through ROS generation [22,23,24,25]. Combined wavelengths provide synergistic benefits. PBM has been applied successfully in wound healing, scar improvement, alopecia, dermatological conditions, musculoskeletal disorders, and chronic pain [19,20,26,27,28]. Laser therapy has been reported to improve urinary incontinence (UI) and pelvic organ prolapse (POP). Women with genitourinary syndrome of menopause and/or SUI treated with PBM report improvements in vaginal dryness, dyspareunia, and incontinence [29,30,31,32,33]. Both lasers and LEDs are considered safe, non-invasive, and drug-free, but differ in coherence, cost, and tissue coverage. Clinical outcomes are dose-dependent, with PBM acting as either stimulatory (tissue repair) or inhibitory (pain relief). Standardized treatment protocols are still lacking, but low-intensity laser and LED devices provide a practical outpatient option for conservative management of PFD [21,22].

Novel dynamic quadripolar radiofrequency (DQRF) devices allow the operator to define the depth and volume of the targeted vulvar area, afford electronic control of movements, and regulate the magnitude of temperature provoked [34]. They generate controlled thermal energy in the range of 40–45 °C, thereby inducing collagen remodeling, angiogenesis, and improved tissue elasticity [34].

Electrotherapy, photobiomodulation, radiofrequency, and electroporation altogether enhance tissue regeneration, restore neuromuscular function, moderate inflammation, and improve vaginal health parameters.

All four options were embodied within the EVA/DAFNE System that was constructed and shaped to deliver personalized multimodal interventions tailored to the specific diagnosis of each patient. The aim of our study was to evaluate the utility, feasibility, safety, and short-term clinical outcomes of customized therapy, via the EVA/DAFNE device, for the treatment of PFD.

## 2. Materials and Methods

Study Design: This was a prospective feasibility cohort study conducted at a tertiary, university-affiliated medical center (San Gerardo Hospital, Monza, Italy) to evaluate the usefulness of multimodal electrical treatment for pelvic floor disorders (PFDs). The study protocol was approved by the local Institutional Review Board (IRB code: GSM-RF 2025).

Participants: Between January and June 2025, women diagnosed with a symptomatic PFD, including POP, SUI, urge or mixed urinary incontinence, voiding dysfunction, pelvic pain, or genitourinary syndrome of menopause, who requested conservative, non-surgical therapy were prospectively enrolled. All patients underwent a standardized gynecological examination performed by one of our staff urogynecologists. Written informed consent was obtained from each participant prior to enrollment.

Exclusion criteria included: age younger than 18 years, active urinary or genital infection, anatomical abnormalities that could interfere with treatment, pregnancy, uncontrolled psychiatric disorders, malignancy, prior pelvic irradiation, or contraindications to energy-based therapies.

Intervention: Participants were treated with the innovative EVA/DAFNE multimodal medical device, which provided customized energy-based therapy based on individuals’ symptoms (see Supplementary Material). The two models, EVA and DAFNE, are complementary and anatomically targeted. Each patient underwent 3 to 5 weekly sessions (depending on the patient’s clinical presentation and response to therapy) administered in the outpatient clinic. No local therapy, anesthesia, or sedation was required. Each session lasted approximately 5–6 min.

All treatments were performed by trained personnel following standardized safety and procedural protocols.

Data Collection: Baseline (before first session) and after-treatment (up to 7 days following last treatment) quality of life and sexual satisfaction were assessed using the following validated scales: Urinary Distress Inventory-6 (UDI-6), Pelvic Organ Prolapse Distress Inventory-6 (POPDI-6), Female Sexual Function Index-6 (FSFI-6), Marinoff Scale, 0–100 visual analog scale (VAS), and Vaginal Health Index. Overall improvement was measured through the Patient Global Impression of Improvement (PGI-I). Each of these questionnaires uses a unique set of questions. Given the cultural and linguistic differences among patients, questionnaires must be appropriately designed to ensure accurate and meaningful results [35]. Among these assessment methods, the VAS is one of the most widely used tools for evaluating pain. It involves a simple scale, ranging from “0” (no pain) to “100” (most severe and unbearable pain). Patients rate their pain levels on this scale, providing a quantitative measure of pain severity. Due to its simplicity and effectiveness, the VAS is a valuable tool for assessing pain responses to treatment.

- The Urinary Distress Inventory-6 (UDI-6) is a validated six-item questionnaire that assesses lower urinary tract symptoms, including incontinence.
- The Pelvic Organ Prolapse Distress Inventory-6 (POPDI-6), a sub-scale of the Pelvic Floor Distress Inventory-20 (PFDI-20), is a six-item composite score that measures prolapse-specific symptoms.
- The Female Sexual Functioning Index FSFI-6 is used to evaluate female sexual response across six domains: desire, arousal, lubrication, orgasm, satisfaction, and pain. The FSFI is a versatile self-report tool that measures the dimensions of female sexual function and consists of 19 questions divided into six domains: desire, arousal, lubrication, orgasm, satisfaction, and pain [17].
- The Marinoff Scale estimates the subjective pain intensity and grades the severity of dyspareunia on a visual analog scale (VAS).
- The 0–100 VAS measures the extent to which the intensity of vulvar pain affects patients’ lives (i.e., “I can’t wear tight-fitting clothing”, “Gets worse when I walk or when I sit”, “I cannot use tampons at all”). The higher the score, the greater the functional limitation. This assessment is conducted for symptoms such as dyspareunia, dryness, irritation/burning, itching, and cytorrhagia.
- The vaginal Health Index score (VHI) encompasses a clinical assessment conducted by a qualified healthcare professional via a speculum examination allowing for the diagnosis of vulvovaginal-atrophy (VVA) after covering five parameters: elasticity, pH, mucosal appearance, moisture, and the presence of vaginal secretion, thereby quantifying the degree of VVA.
- Overall improvement was measured through the Patient Global Impression of Improvement (PGI-I). Subjective improvement is indicated both by “very much improved or much improved” (PGI-I ≤ 2) while non-improvement is indicated both by “minimally improved” or “not improved”.

Outcome: The primary outcome was the feasibility and safety of multimodal electrical treatment for PFD. Secondary outcomes included reported improvement in patient. symptoms and quality-of-life scores.

Statistical Analysis: All statistical analyses were performed. Categorical variables were presented as absolute and relative frequencies, while continuous variables were expressed as mean ± standard deviation or medians with interquartile ranges. Comparisons of non-continuous variables were performed using Fisher’s exact test. A *p*-value of < 0.05 was considered statistically significant. No formal a priori power calculation was performed. However, analysis of the primary outcome, UDI-6 (Urinary Distress Inventory-6), demonstrated a very large effect size (Cohen’s d ≈ 2.2), indicating a substantial improvement in urinary symptoms after treatment and as such supports the adequacy of the sample size for this pilot investigation and provides a strong rationale for future randomized controlled trials.

## 3. Results

Patients (*n* = 26) with a PFD who sought conservative energy-based treatment were enrolled. The cohort consisted of 26 women with a mean age of 48.6 ± 16.7 years. Ten patients (38.5%) had dyspareunia; nine (34.6%) had SUI; six (23.1%) had mild POP; five (19.2%) had genitourinary syndrome of menopause; four (15.4%) had voiding dysfunction; and two (7.7%) had overactive bladder syndrome (Table 1).

Some patients showed significant clinical improvement after only three sessions, while others required up to five sessions. Mean number of treatments per patient was 4 ± 0.8. Table 1 illustrates treatment modalities vis a vis clinical diagnosis.

All scores other than VHI improved (Table 2). Following conclusion of therapy, VAS scores decreased significantly from 72.3 ± 16.3 at baseline to 31.5 ± 21.1 following treatment (*p* < 0.001). UDI-6 scores dropped from 22 ± 8.0 at baseline to 6.6 ± 5.7 following treatment (*p* < 0.001). According to PGI-I scores 88.5% of patients considered themselves improved. Other than mild discomfort during the procedure, no treatment-related adverse effects were noted. PBM therapy delivered via the EVA/DAFNE system proved effective in managing a variety of PFD symptoms and improving quality-of-life scores.

## 4. Discussion

The aim of this study was to evaluate the feasibility and usefulness of the multimodal EVA/DAFNE system, that differentially integrates electrotherapy, photobiomodulation, radiofrequency, and electroporation, for the conservative management of PFDs. We relied on subjective questionnaire surveys and found that the scores obtained from several self-reported validated questionnaires, administered before and after three to five (mean 4 ± 0.8) sessions of LED light therapy including: UDI-6, POPDI-6, FSFI-6, Marinoff Scale, and PGI-I, denoted substantial improvement, and as such the delivery of tailored therapy via the EVA/DAFNE system proved practicable and beneficial.

Although significant perfections were observed in subjective and functional outcomes, the Vaginal Health Index (VHI) did not show a statistically significant change. The VHI reflects structural and morphological changes in the vaginal mucosa, which typically requires longer time to recover compared to the time required for subjective symptoms to manifest. While functional outcomes, such as pain reduction, urinary symptom relief, and sexual function, are punctually influenced by multimodal therapies, other parameters such as elasticity, epithelial integrity, and mucosal thickness get better sluggishly, especially after only three to five sessions. Variability in baseline tissue quality, hormonal status, and menopausal stage may limit detectable VHI changes in this small pilot cohort. The number of sessions parallels a clinical adjustment parameter rather than an analytical variable. All outcome measures were re-collected after completion of the individualized treatment cycle.

Most significant was the improvement in UDI-6 that measures UI and the VAS that evaluates dyspareunia (Table 1). It turns out that combined synergistic interventions can effectively address the heterogeneous manifestations of pelvic floor disorders. As no adverse events were reported, such an integrative approach should be considered tolerable. Management of PFDs should begin with conservative approaches such as lifestyle modification, weight reduction, pelvic floor muscle training, and pharmacotherapy, with more invasive interventions reserved for cases without enough improvement. Yet, physiotherapy techniques that manage pain and quality of life in women with pelvic pain and dyspareunia have limited effects.

PBM provides an effective therapeutic measure, with minimal side effects, for the treatment of lower urinary tract and vulvovaginal symptoms. PBM is practical, safe, painless, well-tolerated, free of immediate or delayed complications, and can be applied on an outpatient basis. The exact pathways by which PBM acts are not yet fully understood. Light is a natural agent consisting of visible and invisible electromagnetic waves that can penetrate soft and hard tissues and induce valuable cellular responses based on the absorption characteristics of the target tissue. LLLT is a fast-growing technology used to treat conditions requiring pain and inflammation relief, healing, and functional restoration. It operates at power levels below those considered hazardous by the FDA and thus avoids therapeutic device regulation and is commercially used for facial skin rejuvenation and wound healing. NIR light may carry different energy levels depending on the intensity of emitted light. Non-thermal NIR wavelengths (850–900 nm), invisible to the naked eye, can activate intracellular cascades of biochemical reactions with local short- and long-term positive effects. Red/NIR light stimulates mitochondria to increase ATP production, enhance gene expression, modulate ROS, and induce transcription factors that trigger protein synthesis, cytokines, growth factors, and inflammatory mediators, leading to cell proliferation and migration [20].

The bulk of the clinical evidence in support of LLLT is based on the effects of laser light at the red/NIR end of the visible spectrum. The biochemical cascades initiated by a single monochromatic (or near-monochromatic) light source may be augmented by the addition of a second light source of a different wavelength and power density. The effects of light of different wavelengths emitted by LEDs have proven versatile and attractive. It appears that an appropriately targeted combination for therapy might yield further clinical benefits. Light-emitting diodes release specific wavelengths tailored to treat various conditions, including neurodegenerative processes, brain pathologies, and rheumatic diseases [26,27,28].

Based on its minimally invasive nature and the lack of significant adverse effects, PBM has emerged as a safe alternative for patients with PFD. PBM is practical and can be applied on an outpatient basis. PBM upregulates antioxidant defense and reduces oxidative stress. It can activate NF-kB in normal quiescent cells. Furthermore, light absorption by ion channels results in Ca^2+^ release, leading to activation of transcription factors, stimulating anti-inflammatory, anti-apoptotic, and antioxidant responses. Yet, the exact pathways by which PBM acts are not yet fully understood.

Differences in PBM effectiveness may be due to parameters such as wavelength, power density, dose, and treatment duration. Optimal stimulation is challenged by the exponential attenuation of penetrating light. Variables including wavelength, spatial coherence, polarity, pulse structure, fluence, treatment duration, and exposure frequency need to be better defined. Further research is needed to determine optimal PBM parameters for PFD. DQRF™ offers an innovative, non-ablative approach by delivering controlled electromagnetic energy to the submucosa, triggering a biological repair cascade that stimulates neocollagenesis and neoangiogenesis. By generating heat through tissue impedance, DQRF reorganizes the extracellular matrix and restores tissue density, elasticity, and hydration, thereby improving GSM, SUI, VVA, and vaginal laxity [34]. Additionally, DQRF provides analgesic effects for pelvic floor hypertonia. Thermal stimulation relaxes overactive muscles, alleviating discomfort, reducing spasms, and improving pelvic function. Enhanced vascularization improves hydration and neuromuscular response, contributing to better sensory function and wellness. DQRF reverses signs of vaginal aging, strengthens connective tissue, and restores mucosal integrity. We have previously shown that non-ablative laser therapy is useful for UI, particularly in older women with vaginal atrophy [29]. Laser therapy is based on the notion that controlled thermal damage stimulates neocollagenesis and angiogenesis, rendering the vaginal walls firmer and tighter [22]. Low thermal energy regenerates vaginal mucosa and restores function in postmenopausal women with GSM and/or VVA [29]. It is essential to trigger desired therapeutic effects while avoiding excessive thermogenesis.

The focus of the study was to assess the efficacy of a *multimodal system* for the improvement of PFD symptoms and patients’ quality of life. We relied on subjective questionnaire surveys and found that the scores obtained from several self-reported validated questionnaires, administered before and after four sessions of LED light therapy including: UDI-6, POPDI-6, FSFI-6, Marinoff Scale, and PGI-I, denoted substantial improvement. Most significant was the improvement in UDI-6 that measures UI and the VAS that evaluates dyspareunia (Table 1). It turns out that combining synergistic interventions can effectively address the heterogeneous manifestations of pelvic floor disorders. As no adverse events were reported, such an integrative approach should be considered tolerable. Management of PFDs should begin with conservative approaches such as lifestyle modification, weight reduction, pelvic floor muscle training, and pharmacotherapy, with more invasive interventions reserved for cases with insufficient improvement. Yet, physiotherapy techniques that manage pain and quality of life in women with pelvic pain and dyspareunia have limited effects.

Based on our short-term pilot study and previous reports, the EVA/DAFNE System emerges as a well-tolerated, minimally invasive, safe, and efficacious treatment for PFD. Rigorous and adequately powered comparative studies are required to investigate PBM benefits. Long-term observational studies will help validate its role in PFD and highlight therapeutic advantages. PBM can be considered an adjunct to other therapies. LED devices are inexpensive and can be incorporated as home-use tools to facilitate compliance. Physicians can rely on these techniques, with clear expectations explained to patients. Existing evidence lacks well-conducted randomized clinical trials and homogeneity of parameters or reproducibility. Scientific evidence from placebo-controlled trials is still scarce. PBM is conducted in multiple ways and uses a versatile combination of parameters, making it difficult to compare results between studies.

The main limitations of this study are the small number of participants, the lack of a control group, and the short evaluation period, which together limit its interpretability and render it insufficient to determine the long-term efficacy and durability of PBM therapy. Additionally, the reliance on self-reported data could potentially introduce bias due to subjective interpretations and reporting inaccuracies by participants. It would be more reassuring if our findings were replicated by others.

Based on this short-term pilot study, LED therapy using the EVA/DAPHNE device resulted efficacious in the treatment of PFD. Multimodal conservative protocols constitute promising treatment alternatives for women with PFDs. Randomized controlled trials minimizing the risk of selection and confusion bias are needed in order to further assess the effect of multimodal BMT on different pelvic floor outcomes and optimize treatment algorithms.

## 5. Conclusions

Energy-based therapy, such as electrotherapy, photobiomodulation, dynamic quadripolar radiofrequency, and electroporation, appears promising as a conservative management option, pending further trials. Our study provides pilot estimates as to the safety and effectiveness of a multimodal integrated treatment protocol for the treatment of PFD. Given the small sample size and lack of a control group, comparative studies involving PFD patients with diversified indications and long-term follow-up are needed in order to further undercover the role of PBM in improving PFDs.

## Figures and Tables

**Table 1:** Indications and treatment modalities.

P PFD	No. (%)	Mean Age	Treatment
Dyspareunia	10 (38.5%)	41.8	EVA
SUI	9 (34.6%)	49.2	2 EVA, 7 DAFNE
Mild pelvic organ prolapse	6 (23.1%)	55	DAFNE
GSM	5 (19.2%)	42.5	EVA
Voiding dysfunction	4 (15.4%)	56	DAFNE
OBS	2 (7.7%)	58.8	DAFNE

Total number of symptoms exceeds the number of patients, as individual patients could report more than one symptom. GSM—genitourinary syndrome of menopause; OBS—overactive bladder syndrome; SUI—stress urinary incontinence.

**Table 2:** Baseline and after-treatment scores: mean ± standard deviation.

	Baseline	Post-Treatment	*p*-Value
UDI-6	22.0 ± 8.0	6.6 ± 5.7	**<0.001**
POPDI-6	13.0 ± 12.8	5.1 ± 9.4	**0.015**
FSFI-6	6.6 ± 7.8	12.9 ± 9.2	**0.010**
Marinoff Scale	1.2 ± 1.4	0.4 ± 0.7	**0.009**
0–100 VAS	72.3 ± 16.3	31.5 ± 21.1	**<0.001**
VHI	16.4 ± 5.3	17.8 ± 5.3	0.339
PGI-I	n/a	2.1 ± 1.0	n/a

In bold all parameters showed significant improvement; UDI—urinary distress inventory; POPDI—pelvic organ prolapse distress inventory; FSFI—female sexual functioning index; VAS—visual analog scale; VHI—vaginal health index; PGI-I—patient global impression of improvement; n/a—non available.

## Data Availability

The data that support the findings of this study are not openly available due to reasons of sensitivity and are available from the corresponding author upon reasonable request.

## Supplementary Materials

The following supporting information can be downloaded at: https://www.mdpi.com/article/10.3390/medicina61122078/s1, additional technical characteristics of the device used in this study, Figure S1: Flow diagram of patient recruitment, inclusion, and analysis. Figure S2: A: Electrotherapy and photobiomodulation probe; B: Radiofrequency+electroporation probe.
