# Supplementary material for: Feasibility of Multimodal Energy-Based Therapy for Pelvic Floor Disorders
Source: Medicina (Kaunas). 2025 Nov 21;61(12):2078. doi: 10.3390/medicina61122078 (PMC12734690; doi:10.3390/medicina61122078)
Supplement: Supplementary file 1 [file medicina-61-02078-s001.zip › medicina-3888634-supplementary.pdf]

Supplementary Materials

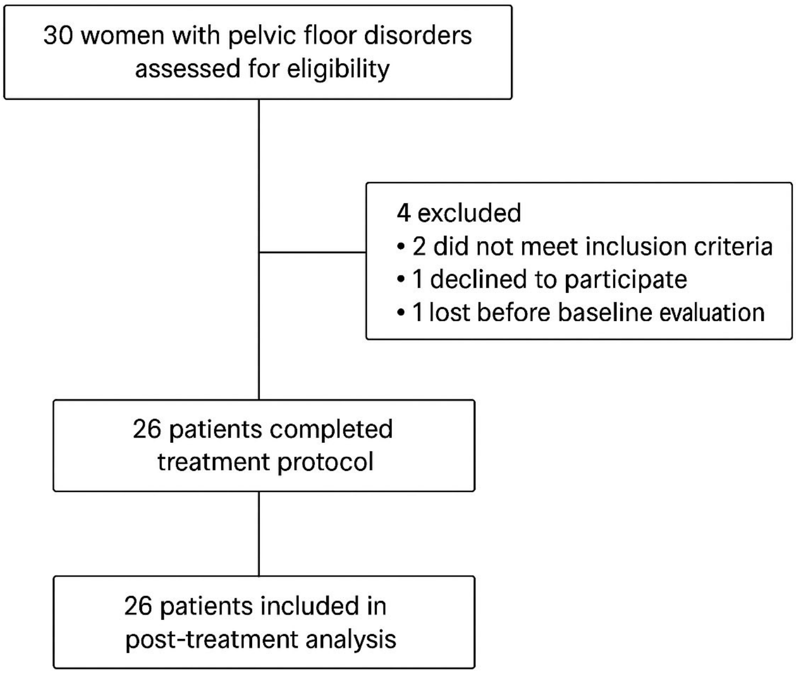

Figure S1. Flow diagram of patient recruitment, inclusion, and analysis.

The EVA/DAFNE System is a 1 MHz radiofrequency generator designed to deliver targeted energy to tissues through a specialized four-electrode applicator. The device was designed and developed by Novavision Group S.p.A., Misinto, Monza-Brianza,Italy. The system inherently integrates four synergistic energy-based technologies (electrotherapy, photobiomodulation, radiofrequency, and electroporation) consenting differentially tailored treatment protocols.

Selected energy combinations and intensities are based on treatment objectives. Electrotherapeutic modalities such as transcutaneous electrical neural stimulation (TENS), Microcurrent Neuromuscular Stimulation (MENS) that uses subthreshold currents of less than 1000 μA) and functional electrical stimulation (FES) are employed to treat PFD by inducing either current analgesia, tissue repair or enhanced muscular functional contraction respectively. Radiofrequency uses controlled thermal energy to induce collagen remodeling and neocollagenesis. Electroporation uses short, high-intensity electrical pulses thereby increasing cell membrane permeability and enhancing the local response.

As regards photobiomodulation, 24 LEDs per wavelength positioned all-round along the entire length of the vaginal probe employ three different wavelengths (UVA 420nm, red 630nm, 870nm NIR/infrared) that diffentially provide antimicrobial, microcirculatory improvement, anti-inflammatory, cellular repair effects.

Dynamic Quadripolar Radiofrequency (DQRF) ensures precise energy delivery by continuously conforming the electrode to focus on the intended tissue layer thereby optimizing tissue remodeling, collagen induction, and pelvic support.

The DQRF technology operates at radio wave frequencies between 1.0 to 1.3 MHz and has a maximum emitting power of 55 watts. Electromagnetic waves in the range of three to six MHz generate electric fields that oscillate at frequencies of two to three thousand per second. DQRF avoid temperature rise beyond 42 C. Combined With Electroporation DQRF incorporates a flat probe ergonomically designed for radiofrequency treatment.

The EVA device was employed for pelvic floor muscles tone. Before each DQRF™ session, the device power was set at 18-20% of its maximum emitting power (10 watts) to reach tissue temperatures of 38-39°C in the target pelvic floor. Each DQRF™ weekly session lasted 6 minutes.

The DAFNE device was employed for the treatment of stress urinary incontinence (SUI). The protocol consisted of two components:

1. Functional Electrical Stimulation (FES) - biphasic square-wave current, frequency 30 Hz, pulse width 250 μs.
2. Dual-wavelength LED photobiomodulation, delivered as:
    - Red light (635 nm): energy density of 8.08 J/cm²
    - Near-infrared light (860 nm): energy density of 5.60 J/cm²

Ultrapulsed Vaginal Electroporation (UPR/EPV) that uses short electric pulses that transiently increases cell membrane permeability and open aqueous channels to facilitate local delivery of drugs, hormones, or nucleic acids.

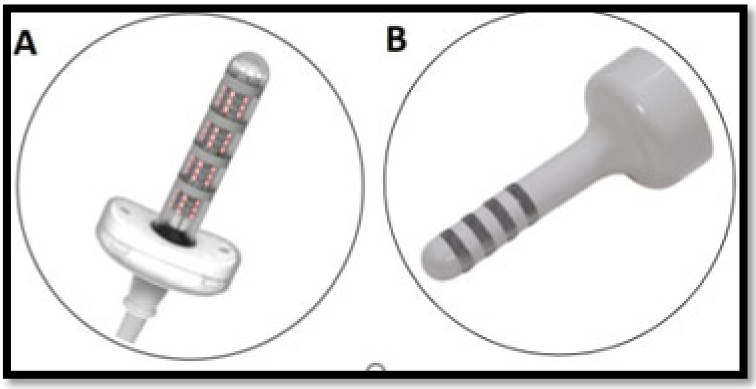

Figure S2. A: Electrotherapy and photobiomodulation probe; B: Radiofrequency+electroporation probe.

The EVA/DAFNE system is equipped with automated software protocols that determine the irradiation sequence and energy intensity according to the selected therapeutic goal and anatomical site. The EVA and DAFNE models are complementary and anatomically targeted. The EVA device operates through VDR (vaginal dynamic radiofrequency) and is therefore primarily used for intra-vaginal applications, such as urinary incontinence, pelvic organ prolapse, genitourinary syndrome of menopause, and vaginal laxity, wherever submucosal thermal remodeling and collagen induction are required. It is designed to automatically adjust and control emission patterns. It automatically adapts radiofrequency emission patterns to minimize unintended energy dispersion. The pulses are

distributed over the vaginal wall, spaced to cover the entire treatment area and are constantly emitted while the handpiece is progressively extracted from the vaginal fundus. The DAFNE device, on the other hand, is designed for external vulvar and perineal applications, targeting dyspareunia, vulvodynia, and post-surgical scarring, wherever photobiostimulation, electroporation, and electrotherapy are required to support epithelial repair and pain modulation. EVA integrates RSS™ (Radiofrequency Safety System) to continuously monitor radiofrequency emissions, temperature variations, and application dynamics. RSS™ measures temperature in real time while simultaneously tracking the movement of the handpiece. This electronic software-driven safety system incorporates multiple sensors. If a drawback is detected, the RSS™ issues a warning and if irregularity persists, automatically interrupts energy delivery.

The vaginal probe has a reusable biocompatible tip that can be sanitized after each use. The emitters are protected by a transparent satin-finished material that allows the propagation of light. Internal and external tips are composed of sterilizable plastic (Radel R5100 NT15) and metal (Stainless steel AISI 316L). The internal tip is made of biocompatible material can be sanitized after each use.

Both devices —the EVA and DAFNE systems—are CE-marked medical devices approved for clinical use in gynecology within the European Union, in accordance with EU Medical Device Regulation (MDR 2017/745). They are classified as Class IIa medical devices, designed for non-invasive functional and regenerative treatments of pelvic floor disorders. Our study was conducted exclusively using these commercially available, certified devices, and no experimental or off-label use was performed.
